# Identification and structural analysis of a carbohydrate-binding module specific to alginate, a representative of a new family, CBM96

**DOI:** 10.1016/j.jbc.2022.102854

**Published:** 2022-12-31

**Authors:** Shiqi Ji, Xuhui Tian, Xin Li, Qunxin She

**Affiliations:** CRISPR and Archaea Biology Research Center, Microbial Technology Institute and State Key Laboratory of Microbial Technology, Shandong University, Qingdao, Shandong, China

**Keywords:** alginate binding, carbohydrate-binding modules, CBM96, multidomain alginate lyase, *Defluviitalea phaphyphila*, CBM, carbohydrate-binding module, DP, degree of polymerization, FN3, fibronectin type III, ITC, isothermal titration calorimetry, M, mannuronate, NCBI, National Center for Biotechnology Information, PDB, Protein Data Bank, SAD, single-wavelength anomalous diffraction, SeMet, selenomethionine

## Abstract

Carbohydrate-binding modules (CBMs) are the noncatalytic modules that assist functions of the catalytic modules in carbohydrate-active enzymes, and they are usually discrete structural domains in larger multimodular enzymes. CBMs often occur in tandem in different alginate lyases belonging to the CBM families 13, 16, and 32. However, none of the currently known CBMs in alginate lyases specifically bind to an internal alginate chain. In our investigation of the multidomain alginate lyase Dp0100 carrying several ancillary domains, we identified an alginate-binding domain denoted TM6-N4 using protein truncation analysis. The structure of this CBM domain was determined at 1.35 Å resolution. TM6-N4 exhibited an overall β-sandwich fold architecture with two antiparallel β-sheets. We identified an extended binding groove in the CBM using site-directed mutagenesis, docking, and surface electrostatic potential analysis. Affinity analysis revealed that residues of Lys10, Lys22, Lys25, Lys27, Lys31, Arg36, and Tyr159 located on the bottom or the wall of the shallow groove are responsible for alginate binding, and isothermal titration calorimetry analyses indicated that the binding cleft consists of six subsites for sugar recognition. This substrate binding pattern is typical for type B CBM, and it represents the first CBM domain that specifically binds internal alginate chain. Phylogenetic analysis supports that TM6-N4 constitutes the founding member of a new CBM family denoted as CBM96. Our reported structure not only facilitates the investigation of the CBM–alginate ligand recognition mechanism but also inspires the utilization of the CBM domain in biotechnical applications.

Alginate is a major component of the cell wall of brown seaweeds (1). It is a linear polysaccharide consisting of residues of α-l-guluronate (G) and β-d-mannuronate (M) in which the two uronic acid epimers are connected by an *α*-(1,4) O-linked glycosidic bond (2). The two sugars are not regularly distributed in the polymer, and they can occur as M-rich and G-rich regions as well as stretches of alternating M and G residues in a long polysaccharide chain (2). As a natural polysaccharide, alginate has been widely used in the food, pharmaceutical, cosmetics, biomaterial, and biorefining industries (3, 4). Alginate oligosaccharides can be generated from alginate lyase–catalyzed β-elimination of alginate polymers. This enzymatic degradation of alginate is biotechnologically important since alginate oligosaccharides and fragments of alginate with DPs (degrees of polymerization) of 2 to 25 exhibit a wide range of biological activities, such as antioxidant, antimicrobial, antihypertension, anticancer (5, 6, 7), and more recently, they were found to enhance the integrity and migration ability of swine small intestine cells (8). To date, a large number of alginate lyases have been characterized, and most of them are derived from marine microorganisms. Fourteen families of alginate lyases are known, including polysaccharide lyase families 5, 6, 7, 8, 14, 15, 17, 18, 31, 32, 34, 36, 39, and 41 according to the CAZy database (database of Carbohydrate-Active enZymes; http://www.cazy.org/) (9).

Carbohydrate-binding modules (CBMs) are often associated with catalytic modules in carbohydrate-active enzymes, including some alginate lyases (10, 11, 12). So far, 91 families of CBMs have been created in the CAZy database (9). These CBMs are of three different types (type A, type B, and type C) based on their features in ligand binding (13, 14): CBMs of type A are capable of interacting with the flat surface of crystalline polysaccharides, those of type B can accommodate individual polysaccharide strand (13). By contrast, CBMs of type C are capable of binding the termini of glycan chains or small sugar molecules, such as a monosaccharide, a disaccharide, or a trisaccharide (13, 14, 15). The most common structure for CBMs is the β-sandwich fold, which is comprised of two stacked β-sheets, each consisting of three to six antiparallel β-strands (13). These CBMs are thought to facilitate the catalysis of the enzymatic degradation of polysaccharides by binding to a carbohydrate ligand. As a result, CBMs show great application potential in different fields of biotechnology, bioprocessing, targeting, cell immobilization, CBM engineering, and CBMs can also be harnessed as analytical tools in bioremediation and modification of fiber (16, 17).

Relatively few CBM families are associated with alginate lyases, and they belong to three families, that is, CBM13, CBM16, and CBM32. These CBMs are reported to have influence on the alginate lyase activity, thermostability, substrate preference, or product distribution (10, 12, 18, 19). Nevertheless, as far as substrate binding is concerned, none of these CBMs exhibit alginate-binding ability. The only known exception is the CBM32 family, which is reported to show a likely type C uronic acid–binding property (18, 20). Recently, we reported that the marine thermophilic bacterium *Defluviitalea phaphyphila* codes for Dp0100, a 201 kDa multimodular and broad-specificity endotype alginate lyase that carries a PL39 family catalytic domain (11). In the present work, we identified a new CBM domain in Dp0100, which exhibits a strong affinity to alginate, and this added another functional domain to this multidomain alginate lyase. Investigation of the CBM–sugar ligand interactions by structural and mutational analyses revealed that the identified CBM domain contains a shallow groove that interacts efficiently with alginate, which represents the first member of a new CBM family.

## Results and discussion

### Dp0100 carries a novel CBM domain responsible for alginate binding

**Figure 1:**
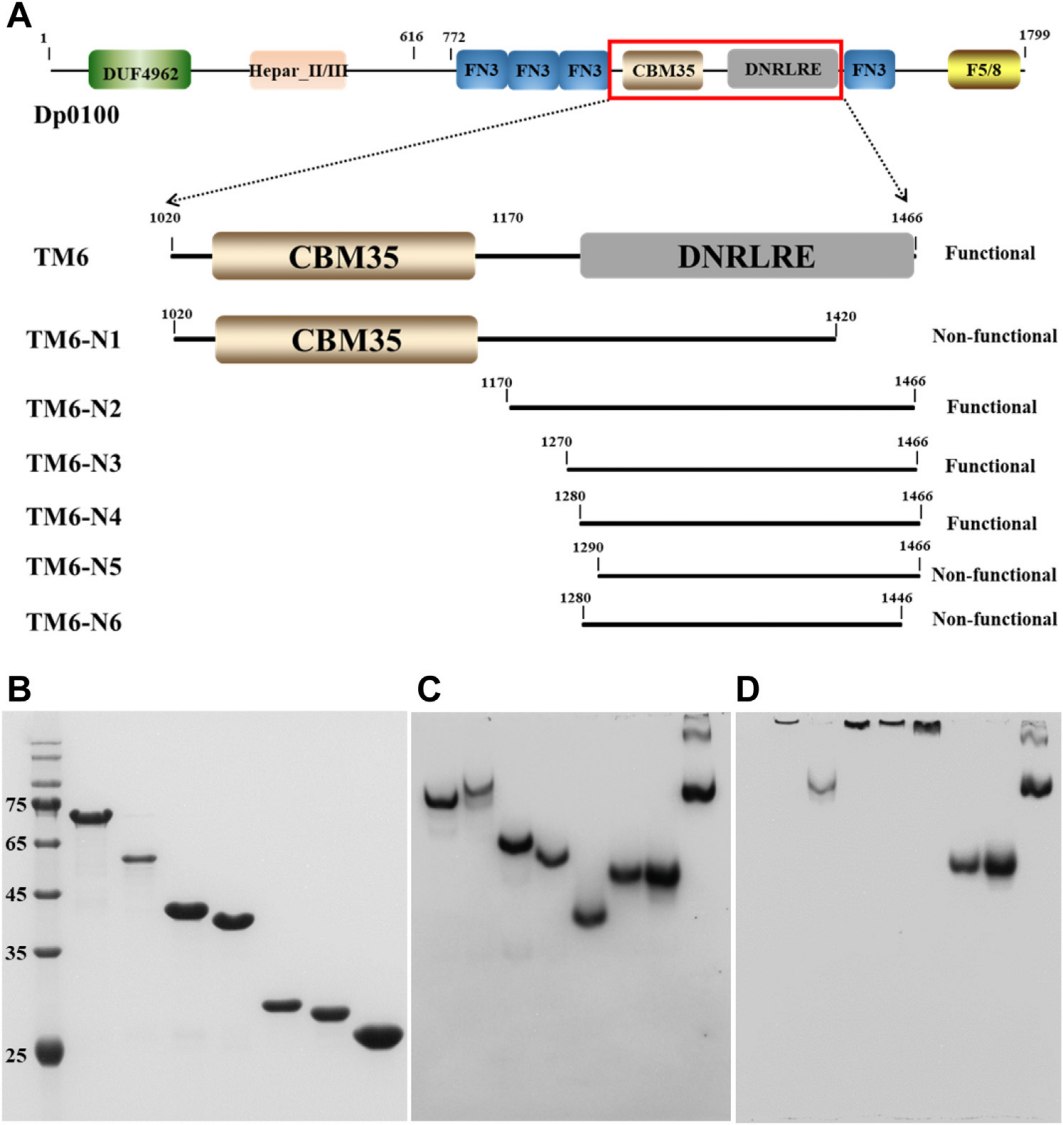
**Schematic diagram and affinity assays for the mutants of Dp0100.***A*, schematic diagram of Dp0100, TM6 fragment, and its truncation mutants. End points of both ends and ends for structural prediction are indicated. *B*, SDS-PAGE of TM6 and its N1–N6 mutants. *C* and *D*, analysis of the affinity of TM6 and its N1–N6 mutants for soluble alginate by the native affinity gel electrophoresis. Native-PAGE without alginate was used as a control in *C*. Native-PAGE in *D* was supplemented with 0.1% (w/v) sodium alginate. Lanes 1 to 8 in *B* represent marker, TM6, and TM6-N1∼N6, respectively. Lanes 1 to 8 in *C* and *D* represent TM6, TM6-N1∼N6, and BSA control, respectively. BSA, bovine serum albumin.

Dp0100 is a multidomain alginate lyase containing eight conserved domains (Fig. 1), among which, two are predicted to be substrate-binding domains, including a CBM35 domain (cd04086) and a CBM32 discoidin domain (also known as an F5/8 type C domain) (pfam00754) (11). A previous protein truncation analysis revealed that the catalytic center is located in DUF4962 and Hepar_II/III domains (Fig. 1), and the same work has also implicated the TM6 segment (from amino acid position 1020–1446) of Dp0100 in alginate binding (11). This region contains a CBM35 domain, which is associated with different carbohydrate-catalyzing enzymes, including β-mannanases, xylanases, α-galactosidases, and glucosyltransferases. Furthermore, the structural domain was found to bind xylan, soluble mannans, mannooligosaccharides, and β-galactan (9, 15). To test if the CBM35 domain in Dp0100 could function in alginate binding, six truncation mutants were designed as illustrated in Figure 1*A*. Their corresponding genes were then cloned to expression plasmids, which were employed for the production of truncation derivatives of TM6 (Fig. 1*B*). The purified proteins were then tested for their interaction with soluble alginate substrate using the native affinity PAGE. The rationale of the binding assay is that the presence of 0.1% alginate in the gel will strongly retard the migration of alginate-binding proteins (11). Interestingly, the binding assay revealed that the mutant TM6-N1 containing CBM35 (Fig. 1*A*) did not show any retardation in the presence of 0.1% alginate compared with TM6 (Fig. 1, *C* and *D*), suggesting that CBM35 does not have the function of alginate binding; by contrast, the migrations of TM6-N2–N4 fragments (Fig. 1*A*) that did not contain any identifiable CBM domain were strongly retarded by alginate (Fig. 1, *C* and *D*). These results indicated that TM6 contains an unknown alginate-binding domain. Furthermore, chopping off 10 amino acids from its N terminus (as in TM6-N5) or 20 amino acids from its C terminus (as in TM6-N6) abrogated the substrate-binding ability of the protein domain (Fig. 1). Thus, the novel alginate-binding domain was defined as the 187 amino acids present in TM6-N4, whereas the upstream CBM35 domain does not play a role in alginate binding. Compared with the three CBM domains (CBM13, CBM16, and CBM32) associated with alginate lyases in the CAZy database, TM6-N4 represents the first CBM domain exhibiting specific alginate-binding activity.

### Isothermal titration calorimetry analysis of TM6-N4 binding to oligoalginate

**Figure 2:**
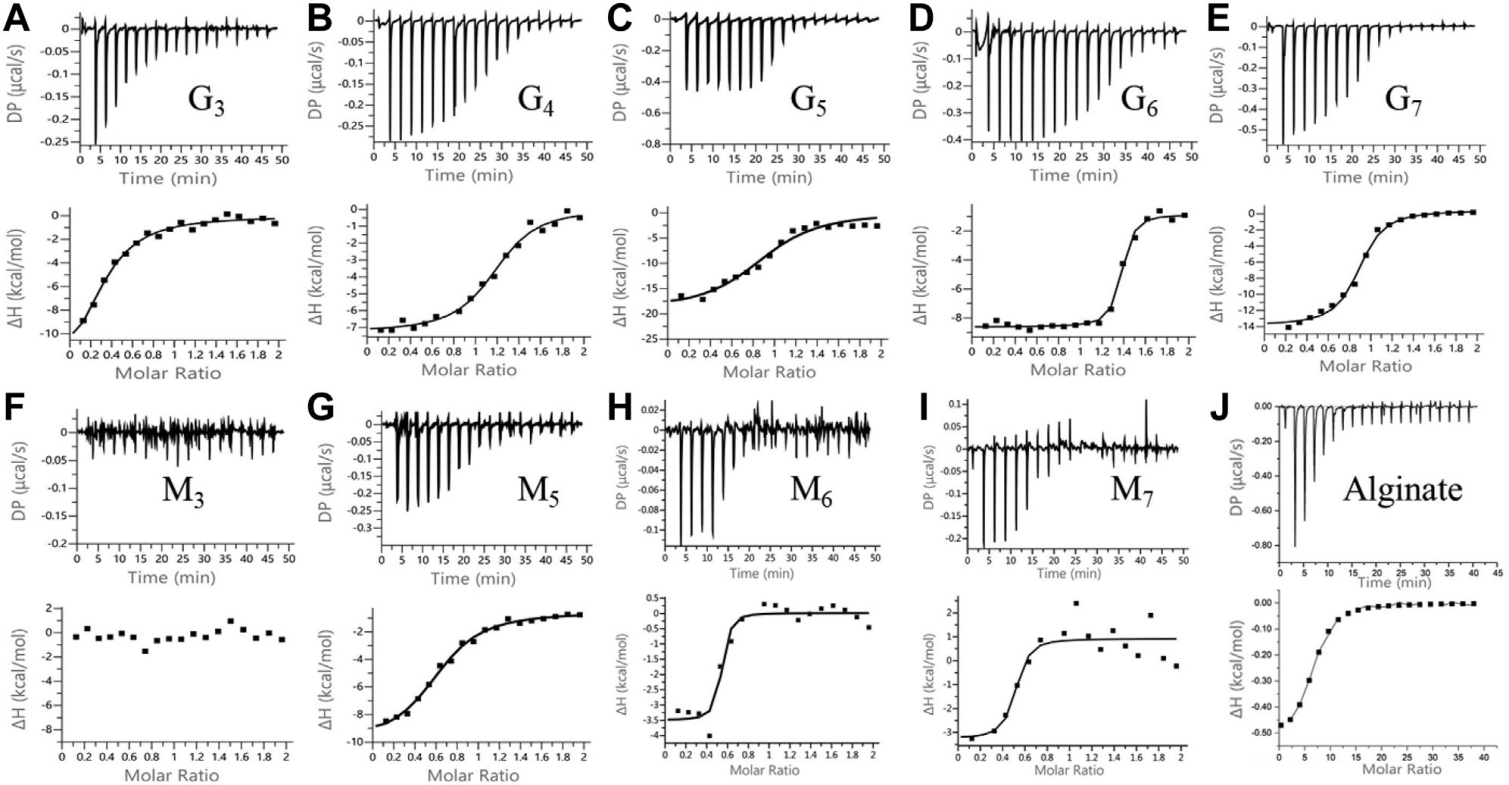
**Thermograms (*upper panels*) and binding isotherms with theoretical fits (*lower panels*) obtained for the binding.** G_3_ (*A*), G_4_ (*B*), G_5_ (*C*), G_6_ (*D*), G_7_ (*E*), M_3_ (*F*), M_5_ (*G*), M_6_ (*H*), M_7_ (*I*), and alginate (*J*) to TM6-N4. The buffer used was 20 mM Tris–HCl buffer, pH 7.0. Protein concentration was 20 μM for TM6-N4.

To characterize the binding of TM6-N4 to alginate, isothermal titration calorimetry (ITC) experiments were carried out at 25 °C and pH 7.0, with alginate and oligoalginate standards including G_3_, G_4_, G_5_, G_6_, G_7_, M_3_, M_5_, M_6_, and M_7_ as substrate and TM6-N4 as the enzyme. As shown in Figure 2, typical ITC thermograms (*upper panels*) and binding isotherms (*lower panels*) with theoretical fits to the experimental data were obtained for the aforementioned oligoalginate standards and alginate. Overall, G oligosaccharides showed an enthalpy-driven binding mode with a slight entropic contribution or penalty, whereas M_6_ and M_7_ showed an entropy- and enthalpy-driven binding mode (Table 1). The results indicated that the binding of G oligosaccharides is dominated by hydrogen bonding and van der Waals interactions, whereas the binding of M oligosaccharides involves both hydrogen bonding and hydrophobic interactions and/or protein conformational changes. The ITC profile obtained for M_3_ (Fig. 2*F*) did not provide reliable thermodynamic data, suggesting that the interaction between TM6-N4 and M_3_ is weak. Shorter alginate sugars (G_2_ and M_2_) failed to yield any reliable thermodynamic data either (data not shown). Nevertheless, G_3_ produced a profile of CBM–ligand interaction (Fig. 2*A*). It is thus likely that the binding sites of TM6-N4 required at least three uronic acid units to yield stable binding complexes. The ITC profile obtained with alginate (Fig. 2*J*) produced the n value of 6.3 and the *K*_*d*_ value of 36 μM, which were much higher than the corresponding values obtained with oligoalginate standards, for example, n = 0.5 and *K*_*d*_ = 0.02 μM with G6 (Table 1). These discrepancies may reflect the fact that one alginate chain has accommodated several protein molecules and the fact of the thermodynamic instability of the long alginate chain. The binding free energy changes for G_3_, G_4_, G_5_, and G_6_ were determined to be −7.5, −8.4, −8.6, and −10.5 kcal/mol, respectively, which increased along with the increase of the substrate chain length (Fig. 2 and Table 1), suggesting the binding groove is long and consists of several subsites for sugar recognition. Moreover, the lowest *K*_*d*_ values were obtained with G_6_ (0.02 μM) and M_6_ (0.06 μM) substrates (Table 1), indicating that TM6-N4 probably forms the most stable complexes with hexasaccharide. Therefore, TM6-N4 may bind individual alginate chains by covering six uronic acid units per molecule and exhibits typical properties of type B CBM.

**Table 1:** Thermodynamic parameters for alginate oligosaccharide binding to TM6-N4 obtained from ITC profiles shown in Figure 2

Ligand	*K*_*d*_ (μM)	Δ*G* (kcal/mol)	Δ*H* (kcal/mol)	*T*Δ*S* (kcal/mol)	N (site)
G_3_	3.1 ± 0.1	−7.5 ± 0.1	−15.1 ± 3.3	−7.6	0.3 ± 0.04
G_4_	0.6 ± 0.1	−8.4 ± 0.1	−7.3 ± 0.2	1.1	1.2 ± 0.02
G_5_	0.5 ± 0.1	−8.6 ± 0.1	−12.3 ± 0.4	−3.6	0.9 ± 0.02
G_6_	0.02 ± 0.02	−10.5 ± 0.1	−8.6 ± 0.4	1.9	1.0 ± 0.02
G_7_	0.3 ± 0.07	−8.9 ± 0.1	−14.2 ± 0.4	−5.4	0.8 ± 0.01
M_3_	No binding				
M_5_	1.2 ± 0.6	−8.1 ± 0.1	−8.6 ± 1.2	−0.6	0.6 ± 0.04
M_6_	0.06 ± 0.06	−9.9 ± 0.1	−3.5 ± 0.3	6.4	0.5 ± 0.02
M_7_	0.2 ± 0.1	−9.3 ± 0.1	−4.2 ± 0.8	5.2	0.5 ± 0.05
Alginate	36 ± 2	−6 ± 0.1	−0.5 ± 0.02	5.5	6.3 ± 0.2

Alginate contains two types of uronate residues (G and M). To compare the binding preference for M or G oligosaccharides of TM6-N4, the binding affinities for pentasaccharides (M_5_ and G_5_), hexasaccharides (M_6_ and G_6_), and heptasaccharides (M_7_ and G_7_) were compared. The binding free energy changes for M_5_ (−8.1 kcal/mol): G_5_ (−8.6 kcal/mol), M_6_ (−9.9 kcal/mol): G_6_ (−10.5 kcal/mol), and M_7_ (−9.3 kcal/mol): G_7_ (−8.9 kcal/mol) (Table 1) are similar, indicating TM6-N4 does not show any apparent binding preference for M or G oligosaccharides.

### Overall structure of TM6-N4 and bioinformatics analysis

**Figure 3:**
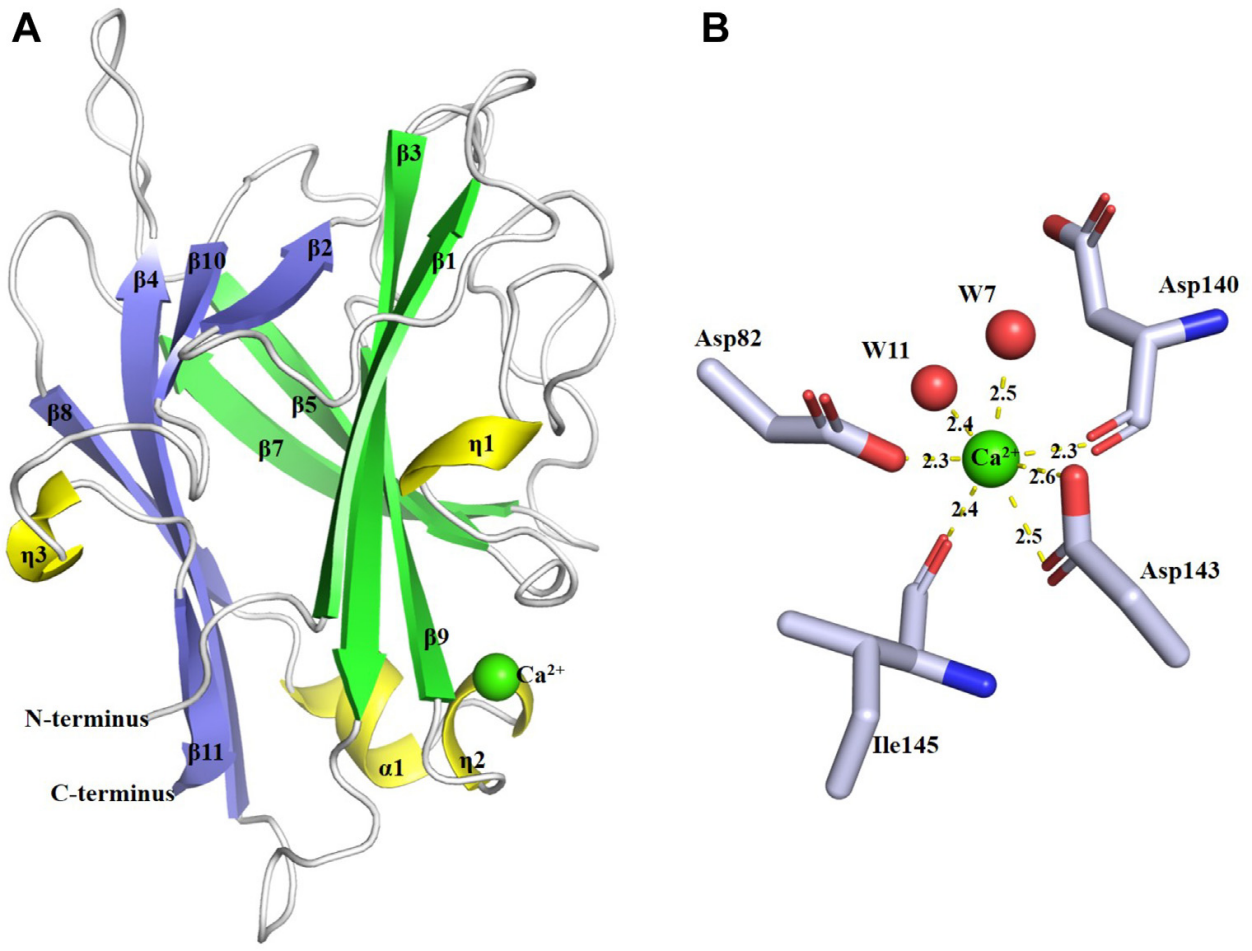
**Structure of the alginate-binding domain of TM6-N4.***A*, schematic diagram of the secondary structure elements of TM6-N4 showing helix as *yellow* and strand as *green* and *blue*. The position of the Ca^2+^ ion is shown as a *green sphere*. *B*, metal-binding site. Amino acid residues (drawn in atom colors [oxygen *red*; carbon *gray*; and nitrogen *blue*]) and water molecules (*red spheres*) in the coordination spheres of the metal ion are shown. *Dotted lines* indicate interactions between metal ion and ligands in the coordinate sphere, and bond lengths are labeled. The figures were prepared using PyMOL.

To gain an insight into the structure–function relationship of this new alginate-binding domain, the TM6-N4 domain protein was expressed in *Escherichia coli*. The purified protein was then used for crystal generation, and the crystal structure of the CBM domain was resolved at 1.35 Å. Structure determination showed that this protein domain is a monomer and an overall architecture of β-sandwich fold (Table 2 and Fig. 3*A*). The single-wavelength anomalous diffraction (SAD) electron density map showed one strong peak in the structure indicating that there is a metal ion in the TM6-N4 structure. Examination of the bond lengths, coordination chemistry, and the nature of the ligands led to the assignment of the peak as Ca^2+^. The Ca^2+^ ion is associated with the strand β9, helix η2, and loops between two β5 and β7. As shown in Figure 3*B*, the metal ion is coordinated by the carboxyl oxygens of Asp82 and Asp142, the main-chain carboxyl oxygens of Asp140 and Ile145, and two water molecules (W7 and W11). Such a location of the Ca^2+^ in TM6-N4 suggests that the metal ion more likely functions in structure stabilization, rather than directly interacting with the target ligand as shown for CBM32 and CBM36 (19, 21). The truncation experiments have shown that the 10 N-terminal residues in TM6-N5 and the 20 C-terminal residues in TM6-N6 significantly influenced the substrate-binding ability (Fig. 1). The overall structure revealed that both segments containing essential secondary structural elements (strand β1 from N terminus and strand β10, helix η2, strand β11 from C terminus) in constituting and sustaining the architecture of β-sandwich fold (Fig. 3*A*).

**Table 2:** X-ray crystallographic data

Data collection	SeMet-TM6-N4
Wavelength (Å)	0.97928
Resolution range (Å)	46.29–1.35 (1.37–1.35)
Space group	*C* 2 2 2_1_
Unit cell (*a*, *b*, *c*) (Å)/(*α*, *β*, *γ*) (°)	31.59, 92.58, 122.26/90.0, 90.0, 90.0
Total reflections/unique reflections	496,701 (16,891)/39,977 (1979)
Multiplicity	12.4 (8.5)
Completeness (%)	99.1 (99.3)
Mean *I*/*σ*	28.1 (2.3)
Wilson *B*-factor (Å^2^)	17.2
*R*_*merge*_*/R*_*meas*_*/R*_*pim*_	0.048 (0.714)/0.050 (0.760)/0.014 (0.255)
*CC*_1/2_	1.000 (0.899)
Anomalous completeness (%)	98.8 (99.2)
Anomalous multiplicity	6.5 (4.4)
Anomalous correlation	0.702 (0.031)
Anomalous signal |DANO|/*σ*(DANO)	1.376 (0.625)


Refinement	
*R*_*factor*_*/R*_*free*_	0.180/0.194
Atoms: protein/ligands/ions/water	2738/5/1/145
Protein residues	182
RMSD (bonds) (Å)/RMSD (angles) (°)	0.0144/1.871
Ramachandran favored (%)/Ramachandran outliers (%)	97/3
Favored rotamers (%)/poor rotamers (%)	98/2
Molprobity score	1.28
Average *B*-factors (Å^2^): main chain/side chains/ligands/ions/water	19.5/22.7/24.5/14.1/28.6
PDB ID	7VBO

**Figure 4:**
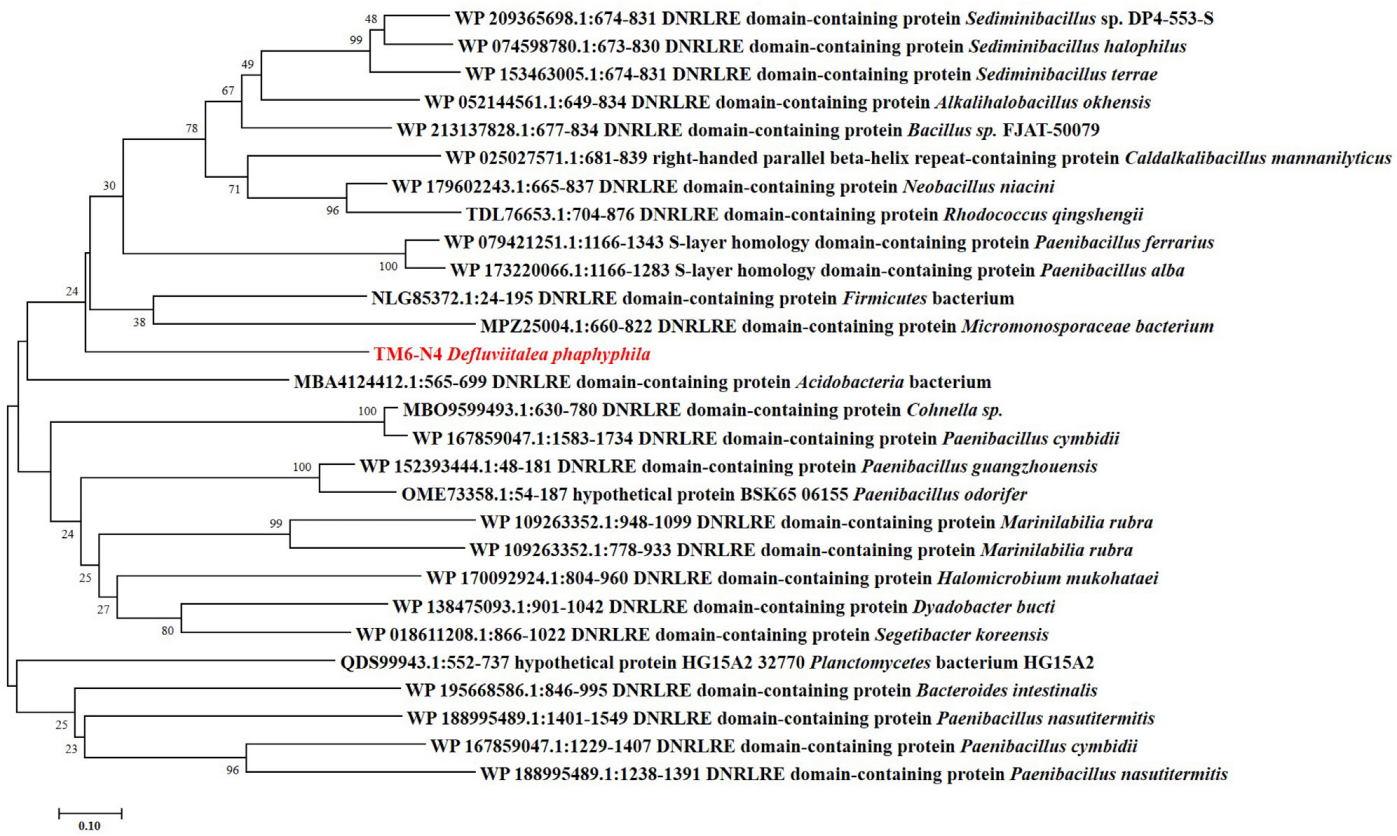
**Phylogenetic analysis of the alginate-binding domain TM6-N4 and its close relatives retrieved from the National Center for Biotechnology Information.** The percentage of replicate trees in which the associated taxa clustered together in the bootstrap test (1000 replicates) is shown next to the branches. The tree is drawn to scale, with branch lengths in the same units as those of the evolutionary distances used to infer the phylogenetic tree.

Both amino acid sequence and structure were submitted to public databases for bioinformatic analysis. Structural comparison using the Dali structural alignment server (22) identified matches between TM6-N4 and various proteins, including transforming growth factor-β family domains (Protein Data Bank [PDB] codes: 4YCG and 5HLY) and those of enzymes involved in the carbohydrate metabolism and related processes (PDB codes: 5EIY, 1PNF, 3WNK, 5X7O, and 2CDO) (Table 3). The aligned regions in these enzymes are normally the accessory CBMs of these proteins (Table 3). Although sequence similarities between these structural domains and TM6-N4 are low, falling into the range of 6 to 12% identity, their RMSD values (2.7–3.3 Å) are small (Table 2), which implies that these structural domains have a strong propensity to adopt similar folds and thereby function in substrate binding. Protein BLAST search in the GenBank databases using the TM6-N4 sequence revealed that the novel CBM domain showed ca. 30% sequence identity to the members of the DNRLRE domain (National Center for Biotechnology Information [NCBI] conserved domain accession no.: NF033679) (Figs. 1*A* and 4). DNRLRE domain was first characterized by the sequence motif (DNRLRE) occurring in one or more times in the proteins of archaeal disaggregatase, S-layer proteins, and so on (23), and their functions remain unknown. Our blast results also showed that DNRLRE domain is present in some carbohydrate-degrading enzymes, for example, alginate lyase from *Paenibacillus* sp. JC52 (NCBI accession no.: WP_144851692), hyaluronate lyase from *Halomicrobium* sp. LC1Hm (NCBI accession no.: QGA84466), and pectate lyase from *Rhodococcus qingshengii* (NCBI accession no: TDL76653). Analyses of these sequences by Clustal Omega (EMBL-EBI) alignments (24) revealed quite a few conserved amino acids/sequence motifs (Fig. S1). To gain an insight into the possible function of these evolutionary conserved amino acids, ConSurf server (https://consurf.tau.ac.il/consurf_index.php) (25, 26, 27) was employed to map evolutionary conservation sites onto TM6-N4 structure (Fig. S2). The results showed that most of these conserved sites are the residues with hydrophobic side chains (*e.g.*, Val7, Ala19, Ile37, Phe42, Ala54, Trp84, Ile131, Ile135, Ala147) distributing in the β-sheets and helixes of TM6-N4 structure (Fig. S2). Thus, these conserved sites are believed to sustain the secondary and β-sandwich architecture through hydrophobic interactions. It is also found that Lys27 and Arg36, which are later proven as key residues in ligand recognition, are relatively conserved in TM6-N4 structure. Together, the aforementioned data suggest that these domains can form a novel CBM family, and a family name of CBM96 was assigned according to the CAZy database (9).

**Table 3:** Structural comparison with TM6-N4 using the Dali server

PDB code	Z score	RMSD (Å)	LALI^a^	N_RES_	Identity (%)	Function	Reference
4YCG	10.7	3.1	132	198	12	Bone morphogenetic protein 9 growth factor domain	(54)
5HLY	9.9	3.1	117	299	14	Proactivin A precursor	(55)
5EIY	8.5	2.7	111	658	12	Cellulose synthase	(56)
1PNF	8.3	3.1	108	314	10	Oligosaccharide recognition residues of peptide-*N*(4)-(*N*-acetyl-β-d-glucosaminyl) asparagine amidase	(57)
3WNK	8.2	2.8	107	712	6	CBM35 in cycloisomaltooligosaccharide glucanotransferase	(58)
5X7O	8.2	3.1	114	1247	8	CBM35 in α-1,6-glucosyltransferase	(59)
2CDO	8.0	3.3	115	138	9	CBM6 in β-agarase	(60)

a Total number of equivalent residues.

### Alginate-binding site analysis

**Figure 5:**
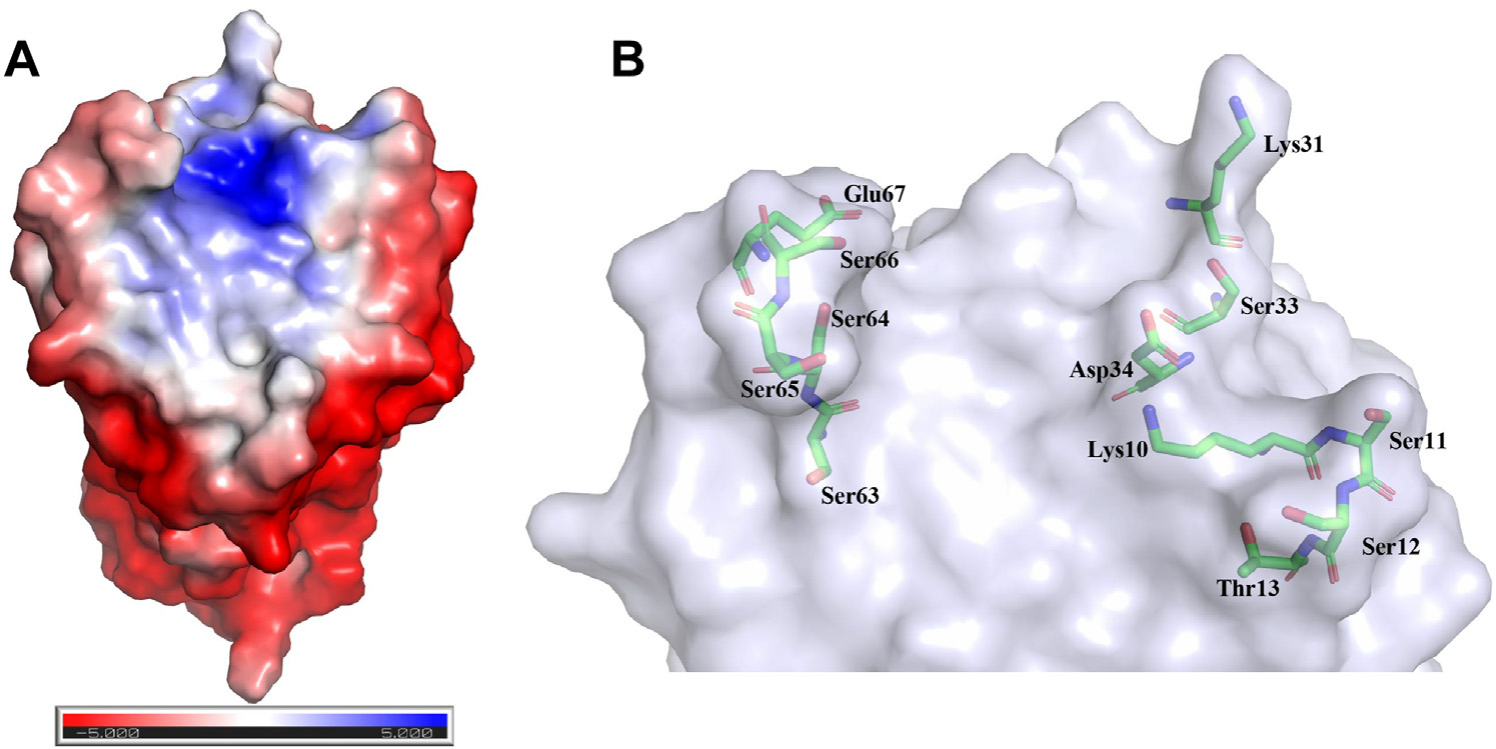
**Surface electrostatic potential of TM6-N4 and surface representation of its binding walls.***A*, the surface electrostatic potential of TM6-N4 contoured at 5 kT/e. A positive potential (*blue*) appears in the suggested substrate-binding groove at pH 6. *B*, surface representation of the walls of the cleft of TM6-N4 to show constituted residues. Wall residues are highlighted (drawn in atom colors [oxygen *red*; carbon *green*; and nitrogen *blue*]) with the remainder of the enzyme shown transparently in a surface format in *B*. The figures were prepared using PyMOL.

To define the alginate-binding site in TM6-N4, its electrostatic surface potential was calculated using the APBS-PDB2PQR software suite (https://server.poissonboltzmann.org/) (28). Using the crystal structure as input, an overall positively charged electrostatic binding groove was generated at pH 6 (Fig. 5*A*). This is consistent with the occurrence of several positively charged amino acids in this region, including lysine and arginine residues. In addition, amino acids with hydrophilic polar side chains like serine, asparagine, threonine, and glutamine are also enriched around the predicted binding site. A broad and shallow groove is positioned within a loop region of the β-sheets (Fig. 5). On one wall of the groove, there are four conjoint serines (Ser63–66) and Glu67, whereas seven other polar amino acids, that is, Lys10, Ser11, Ser12, Thr13, Ser30, Ser33, and Asp34, are present on the opposite wall (Fig. 4*B*). These polar residues have a strong propensity to form extensive hydrogen bonds with the carboxyl and hydroxyl groups of alginate ligands and thereby driving the CBM–alginate interactions.

**Figure 6:**
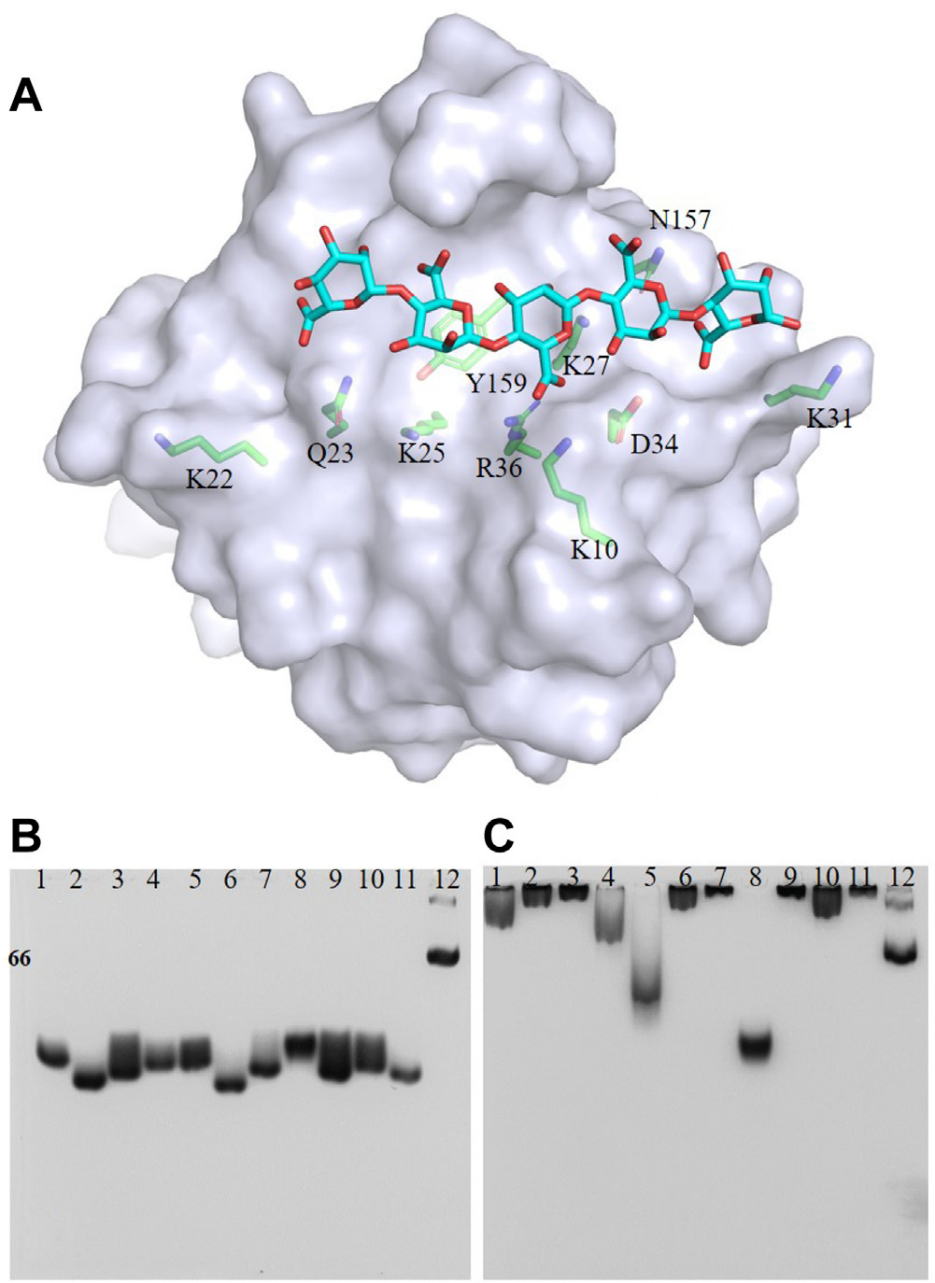
**Key residues revealed by docking and affinity assays for the site-directed mutants.***A*, surface representation of a docked pentasaccharide M_5_ in the cleft of TM6-N4 to show the binding site (ligand shown as *sticks* and drawn in atom colors [oxygen, *red* and carbon, *cyan*]). Key residues interacting with the substrates are highlighted (drawn in atom colors [oxygen, *red*; carbon *green*; and nitrogen, *blue*) with the remainder of the enzyme shown transparently in surface format. The figure was prepared using PyMOL. *B*, native PAGE without alginate of TM6-N4 and its site-directed mutants served as a control. *C*, affinities of TM6-N4 and its site-directed mutants for soluble alginate by native affinity gel electrophoresis. Lanes 1 to 2 represent K10A, K22A, Q23A, K25A, K27A, K31A, D34A, R36E, N157A, Y159A mutants, TM6-N4, and BSA, respectively. BSA, bovine serum albumin.

Thus, we reasoned that the alginate-binding site of TM6-N4 lies in the shallow and broad groove. The length of the binding groove is in good accordance with the ITC results in which six subsites can be accommodated. Then, we attempted to generate cocrystals of the TM6-N4 CBM–alginate substrates. Extensive cocrystallization and soaking attempts were made by employing oligosaccharides of G_3_ to G_7_ or M_5_ to M_7_, in combination with different crystallization conditions and point mutants of the CBM domain, but all these attempts failed to yield any cocrystals. Hence, to study the binding interactions of TM6-N4 with an alginate oligosaccharide, we docked a mannuronate pentasaccharide (M_5_) in the predicted binding site. The ligand M_5_ has 13 active torsions, preventing the docking program from producing any accurate model. To reduce the calculation complexity, we reduced the active torsions to seven by analyzing the torsions of mannuronate oligosaccharide ligands deposited in the PDB. M_5_ was found to form different docking poses in the extended groove, and the binding free energies are quite close (−7.5 ∼ −7.1 kcal/mol). The ligand goes through the course of the groove with the receptor then presented (Fig. 6*A*). The resulting ligand conformation reveals the potential importance of several residues (Lys10, Gln23, Lys25, Lys27, Arg31, Asp34, Arg36, Asn157, and Tyr159) in substrate recognition (Fig. 6*A*).

**Figure 7:**
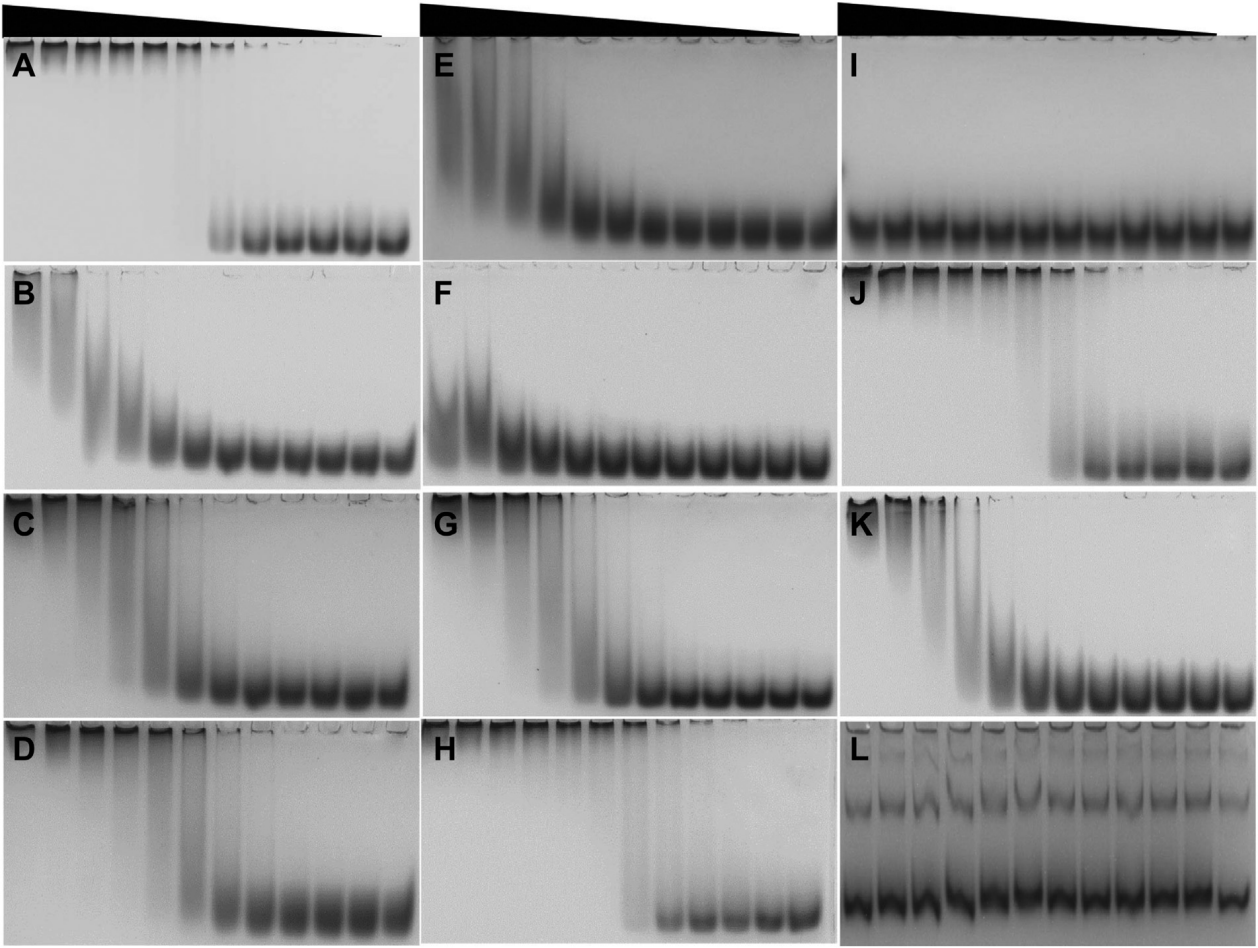
**EMSA interaction between alginate and site-directed mutants of TM6-N4.** Decreasing concentrations (0.6 to 0.0006%, w/v) of alginate were incubated with different mutants of TM6-N4. TM6-N4 (*A*), K10A (*B*), K22A (*C*), Q23A (*D*), K25A (*E*), K27A (*F*), K31A (*G*), D34A (*H*), R36E (*I*), N157A (*J*), Y159A (*K*), and BSA (*L*). The *black wedge* indicates the decrease in alginate concentration. Lane 1, 0.6%; lane 2, 0.3%; lane 3, 0.15%; lane 4, 0.08%; lane 5, 0.04%; lane 6, 0.02%; lane 7, 0.01%; lane 8, 0.005%; lane 9. 0.0025%; lane 10, 0.0012%; lane 11, 0.0006%; and lane 12, blank control without alginate. BSA, bovine serum albumin.

To figure out the key residues in alginate recognition, site-directed mutagenesis was conducted for the amino acids revealed from the electrostatic surface potential calculation and dock modeling. Ten TM6-N4 mutants were obtained, including K10A, K22A, Q23A, K25A, K27A, K31A, D34A, R36E, N157A, and Y159A (Fig. S3). These mutant proteins were then tested for alginate-binding ability using the native affinity PAGE (12%, w/v). As shown in Figure 6, *B* and *C*, the mutant R36E migrated similarly regardless of whether 0.1% (w/v) alginate was present in the gel or not. The single mutation of R36E can cause complete abortion of alginate-binding ability, indicating Arg36 plays a central role in alginate recognition, whereas most of the other mutants were retarded to the top of the gel. To make further discrimination of the contributions of the mutated residues to alginate binding, an EMSA experiment was carried out. As shown in Figure 7, proteins showed different migration profiles as the changing of alginate concentrations. The mutants of Q23A (Fig. 7*D*), D34A (Fig. 7*H*), and N157A (Fig. 7*J*) showed a similar migration profile as the wildtype (Fig. 7*A*), indicating the mutation had little influence on alginate-binding ability, whereas R36E (Fig. 7*I*) showed a similar migration profile as the bovine serum albumin control (Fig. 7*L*), indicating the alginate-binding ability was deprived by the mutation. Substantial protein shifts in lane 1 (0.6% alginate) were observed in Figure 7*B* (K10A), Figure 7*E* (K25A), and Figure 7*F* (K27A), confirming their key roles in alginate recognition. The mutants of K22A (Fig. 7*C*), K31A (Fig. 7*G*), and Y159A (Fig. 7*K*) also showed remarkable influences on the alginate-binding ability, and substantial protein migrations occurred in lane 3 (0.15% alginate). These results indicated that the amino acids located in the bottom or the wall of the shallow groove, including Lys10, Lys22, Lys25, Lys27, Lys31, Arg36, and Tyr159, are essential for alginate binding. Their positive side chains (Lys10, Lys22, Lys25, Lys27, Lys31, and Arg36) or the planar of aromatic ring (Tyr159) are speculated to mediate CBM–ligand recognitions. Other polar or negatively charged residues such as Gln23, Asp34, and Asn157 are not important for alginate recognition. It is reported that hydrophobic stacking interactions between aromatic residues and ligands often play key roles in type B CBM-glycan binding (29). The binding mode of TM6-N4 to alginate is different from those reported for CBMs specific to glycan ligand, which possibly attributes to the soluble and negatively charged nature of alginate.

To test if the loss of substrate binding could be resulted from misfolding of the mutant proteins, we performed CD spectroscopy measurement. As inferred from CD spectroscopy, the secondary structures of all mutants are similar to that of the wildtype (Fig. S4). Thus, we conclude that the loss of alginate-binding property results from the mutation in the binding groove.

### Biological and biotechnical significance analysis

TM6-N4 is originated from Dp0100—the largest alginate lyase ever reported (11). We attempted to solve the structure of Dp0100 using crystallization and cryo-EM methods, but attempts constantly failed, which could be attributed to the flexibility of macromolecule. Indeed, in our previously reported negative-stain electron microscopy trial (11), the homogeneity of the protein particles is unsatisfactory, indicating the conformation of Dp0100 is probably not stable. With the acquired structure knowledge of the catalytic domain (PDB code: 6JP4) and TM6-N4 domain of Dp0100, we attempted to generate the multimodular structural features of Dp0100 by using RoseTTAFold, a deep learning–based prediction method (30). As the full length of Dp0100 (1799 amino acids) exceeds the upper limit (1500 amino acids) of the program, the peptide of 616 amino acids was removed from the N terminus of Dp0100, giving the truncated 1183 amino acid residues (Dp0100-1183, Ala617–Gln1799) that were submitted for structure prediction. As shown in Figure 1*A*, the catalytic domain of Dp0100 is located between Ala1 and Val772. Therefore, Dp0100-1183 still retained 156 amino acids of the catalytic domain of Dp0100, and doing so would facilitate the construction of the overall structure of the full-length protein. As shown in Fig. S5, the noncatalytic modules of Dp0100 showed a ribbon pattern, which is inconsistent with the high flexibility prediction of the macromolecule. Six recognizable fibronectin type III (FN3) modules are presented in the predicated Dp0100 structure. Typically, FN3 modules adopt a characteristic β-sandwich fold of three antiparallel β-strands atop four antiparallel β-strands (31). At present, the functions of these FN3 modules remain enigmatic. Nevertheless, based on their spatial conformations, they show a strong propensity to increase intermodular flexibility, and they may also function in the recruitment of other enzymes to facilitate alginate degradation (31). It is reported that one of the physiological roles of these sugar-binding functional CBMs helps the enzymes contact with carbohydrates and increases the concentration of the enzymes in the vicinity of the substrate and then facilitate polysaccharide degradation (32). As reported in our previous work (33), *D. phaphyphila* Alg1 as a brown algae–degrading strain, the evolution of efficient systems for utilizing the cells of brown algae is vital for its survival. The appearance of the unusual modularity complexity (Fig. S5) and substrate-binding ability in Dp0100 is likely the consequence of long-term evolution and adaption in a coexistence environment of brown seaweed.

The identification of TM6-N4 as the first alginate-binding CBM has important implications in research of alginate synthesis and degradation as well as in biotechnological applications of alginates and their derivatives. Taking the advantage of alginate recognition, the CBM can be utilized for studying brown algae cell structure and alginate synthesis, like xylan-specific CBMs were used for the recognition of plant cell walls (34). Because alginate can also be produced by the pathogenesis of *Pseudomonas aeruginosa* (35), alginate-specific CBM can also be used for the design of potentially pathogenic bacteria detection tool. Moreover, enzyme engineering could be conducted by fusion of the alginate-specific binding CBM to a well-characterized alginate lyase in order to significantly increase or radically change enzyme activities/specificities.

## Conclusion

In this work, we identified the first alginate-binding module TM6-N4 present in the multimodule alginate lyase Dp0100. Structural and mutational analysis revealed a long-extended binding groove for the alginate chain, characteristic of type B CBM fold. ITC and docking results indicated that the binding groove consists of six subsites for sugar recognition. Crystal structural analysis and biochemical characterization showed that amino acid residues located in the bottom or the wall of the shallow groove, including Lys10, Lys22, Lys25, Lys27, Lys31, Arg36, and Tyr159, are essential for alginate binding. A new CBM family of CBM96 was created based on our characterization. The discovery of the first alginate-binding domain in Dp0100 has yielded new insights into the mechanisms of CBM–alginate recognition as well as the potentials of alginate-specific CBM utilizations.

## Experimental procedures

### Cloning, overexpression, and purification

Strain *D. phaphyphila* Alg1 and alginate lyase Dp0100 have previously been reported (11, 33). Heterologous expression and purification of Dp0100-TM6, its truncated derivatives, and the site-directed mutants were conducted by following the methods described previously with minor modifications (11). For cloning, the gene was amplified, purified, and ligated into pEASY-E1 (TransGen Biotech, Inc), and the primers are described in Table S1. The resulting plasmids were then transformed into *E. coli* DH5*α* cells. Single colonies were picked from the plate and cultured in ampicillin LB medium. After sequence verification, the recombinant plasmids were transformed into *E. coli* BL21 (DE3). Strains for protein expression were grown in fresh ampicillin LB medium. After growth to an absorbance of 0.5 to 0.6 at 600 nm, IPTG was added to a final concentration of 1 mM, and the temperature was incubated at 25 °C for an additional 12 h. For protein purification, the cells were harvested and then resuspended in the binding buffer (50 mM Tris–HCl, 200 mM NaCl, pH 8.0) and ruptured by a cell disruptor. The supernatant was then heated at 60 °C and incubated for 10 min to precipitate *E. coli* proteins. The precipitant was removed by centrifugation as aforementioned, and cell-free extract was applied on a 5 ml HisTrap cartridge (Cytiva). Proteins were eluted by a 50 ml gradient of imidazole concentration from 0 to 300 mM at a flow rate of 2 ml/min. Elution fractions were collected and analyzed by SDS-PAGE. Protein was then concentrated to a volume of 1 to 2 ml using a VivaSpin concentration device of 10,000 molecular weight cutoff. The resulting recombinant protein was further purified by size-exclusion chromatography using Superdex 200 Increase 10/300 GL column (Cytiva). Chromatography was performed on an ÄKTA purifier system (Cytiva). Various physical and chemical parameters of the expressed proteins such as molecular weight, theoretical pI (isoelectric point), amino acid composition, and extinction coefficient were obtained by using the web tool ProtParam (https://web.expasy.org/protparam/) (36). Protein concentrations were measured by using Nanodrop 2000 spectrophotometer (Thermo Fisher Scientific). Truncated mutant information from TM6-N1 to TM6-N6 is listed in Figure 1*A*.

### Affinity gel electrophoresis and EMSA

To evaluate the binding ability of Dp0100-TM6 and its truncated mutants to soluble polysaccharide alginate, affinity gel electrophoresis was employed (11). Sodium alginate from brown algae (viscosity >0.02 Pa·s, 1% alginate in H_2_O at 25 °C) was purchased from Sangon Biotech (Shanghai) Co Ltd. Alginate was added at a concentration of 0.1% (w/v) into the separation gel. Gels without the addition of alginate and bovine serum albumin were used as negative controls. Moreover, a modified EMSA method (37) was used to evaluate the influences of site-directed mutations on protein–alginate interactions. Site-directed mutants with a concentration of 0.2 mg/ml were mixed with different concentrations of alginate (0.6–0.0006%, w/v) in a buffer of 20 mM Hepes (pH 7.5) and 50 mM NaCl. After incubation for 10 min at 50 °C, samples were loaded for native PAGE. Electrophoresis was carried out at room temperature in native 12% (w/v, affinity gel electrophoresis) or 8% (w/v, EMSA) polyacrylamide gels. After electrophoresis, proteins were visualized through staining with Coomassie blue.

### ITC

The proteins were dialyzed extensively against buffer with 20 mM Tris–HCl (pH 7.0) without NaCl and adjusted concentration to 20 μM, and the oligosaccharide ligands (purity ≥95%; Qingdao BZ Oligo Biotech Co Ltd) or alginate ligands (viscosity >0.02 Pa·s, 1% alginate in H_2_O at 25 °C) were dissolved in the same buffer to minimize heats of dilution with a concentration of 200 μM. To avoid bubble formation, all the samples were degassed by centrifugation at 12,000 rpm/10 min. Calorimetric titration was performed with MicroCal PEAQ-ITC (Malvern Panalytical). During a titration experiment, the protein sample was stirred at a speed of 750 rpm and kept a constant temperature of 25 °C, and the titration needle was filled with 80 μl oligosaccharide or alginate ligand. Titrations were completed after 19 injections (each 3 μl) at an interval of 150 s. The data were analyzed by using the one-site binding model in the MicroCal PEAQ-ITC Analysis software. The fitted data yielded the dissociation constant (*K*_*d*_), the number of binding sites on the protein (n), and the enthalpy of binding (*ΔH*). Other thermodynamic parameters can be calculated by using the standard thermodynamic equation *ΔG* = −RTln *K*_a_ = *ΔH*-T*ΔS*. Titrations were carried out in triplicate for most ligands, and the errors are the SD of the mean of these replicates.

### Phylogenetic analyses

The amino acid sequence with the minimum functioning length for alginate binding (TM6-N4) was submitted to NCBI nonredundant protein sequences database for standard protein BLAST (38). Representative amino acid sequences with high identities with TM6-N4 were retrieved from the database. These sequences were aligned by Clustal Omega (24), and phylogenetic analysis was performed using the software package MEGA version 7.0 using the neighbour-joining method (39).

### Crystallization and data collection

Initial crystallization conditions were determined by automated screening (NeXtal, Qiagen, Inc) using a Matrix Hydra II crystallization robot. Crystals of selenomethionine (SeMet)-labeled TM6-N4 construct were optimized by hanging-drop vapor diffusion using a 1:1 ratio of protein to precipitant. In detail, 30 mg/ml protein in 10 mM Tris–HCl (pH 8.0) was added to the same volume of precipitant. A precipitant containing 1.8 M lithium sulfate and 0.1 M Tris–HCl (pH 8.0) was used. Crystals were formed after equilibrating against a 1 ml reservoir of the same precipitant over the course of 1 day at 16 °C. For the cocrystallization with a substrate, 5 mM ligand was added to a solution containing the construct TM6-N4. Oligosaccharides of G3 to G7 or M5 to M7 (Qingdao BZ Oligo Biotech Co Ltd) were used as ligands for cocrystallization and soaking. All crystals were cryoprotected in 100% glycerol, prior to flash cooling in liquid nitrogen, and X-ray diffraction data were collected at the Shanghai Synchrotron Radiation Facility on beamline BL19U1.

### Phasing, structure determination, and refinement

Crystallographic phases were determined using SAD data collected from a crystal of SeMet-labeled protein. SeMet peak SAD data were collected at 0.97928 Å wavelength on beamline BL19U1 at the Shanghai Synchrotron Radiation Facility. Data were processed in autoPROC (Global Phasing Limited) (40, 41, 42, 43, 44, 45) to 1.35 Å, in spacegroup *C*222_1_. The SHELX program suite (46) was used to identify heavy atom sites and produce an initial electron density map. A preliminary model was built using Coot and Buccaneer (47, 48) and subsequently subjected to rounds of building in Coot. The overall structure was then iteratively refined using REFMAC-5 (49). The final model includes residues from −2 to 179 of the expected 204 residues (187 from the protein and 17 from the N-terminal His tag). Some additional weak electron density could be seen for each subunit at the C terminus arising from residues from the His tag. These were not modeled in the structure. Refinement statistics are summarized in Table 1. The model was validated using MolProbity (50), and diagrams were generated using PyMOL (Schrödinger) (51). The coordinate of the final structure was deposited in PDB (Table 1).

### Site-directed mutagenesis

Site-directed mutagenesis was conducted by designing a pair of complementary mutagenic primers to amplify the entire plasmid in a thermocycling reaction with a high-fidelity *pfu* polymerase (New England Biolabs, Inc). The nucleotide sequences of the mutagenic primers used for mutagenesis are given in Table S1. The PCR product was digested with DpnI (New England Biolabs, Inc) at 37 °C for 1 h to degrade the parental plasmid DNA. The product from the DpnI digestion was transformed into *E. coli* BL21 (DE3) competent cells. The cells were spread on LB plates containing 100 μg/ml of ampicillin and incubated at 37 °C overnight. Single colonies were inoculated in 5 ml of ampicillin LB medium and cultured for 12 h. The plasmids were extracted from the recombinant *E. coli* cells, and the inserts were sequenced to confirm the presence of the desired mutation. The truncated protein was produced and purified in the same way as described previously.

### CD spectroscopy

CD spectra of wildtype and mutants of TM6-N4 were measured at 25 °C with a Jasco J-1500 CD Spectrophotometer using a quartz cuvette with a path length of 1 mm. Protein samples were measured in H_2_O to a concentration of 0.2 mg/ml in a total volume of 400 μl. The measurements were recorded at wavelengths from 260 to 190 nm with continuous scanning (100 nm/min) using a bandwidth of 1 nm, data pitch of 0.1 nm, and with a total of three scans for each. Data were collected considering the HT voltage applied to the detector was under the 600 voltage. The spectra were corrected by subtracting the spectrum from the H_2_O background.

### Docking simulation

To study the binding and interactions of TM6-N4 with alginate, we docked an M_5_ as a ligand in the presumed binding site. Its structure was obtained from the crystal structure of TM5 H187A mutant complexed with M_5_ (PDB code: 6JPN) (11). Docking simulations were conducted using AutoDock MGL tools and AutoDock Vina to prepare the systems for calculations (52, 53). A grid box was created to cover the sugar-binding region estimated from the structural analysis, assuming the protein was rigid and the ligand was flexible. The selected structure was superimposed using PyMOL, version 2.4 (51).

### Accession number

The X-ray crystal structure for the alginate-binding domain TM6-N4 and the associated X-ray data have been deposited in the PDB under the ID code 7VBO.

## Data availability

The atomic coordinate and structure factor (PDB code: 7VBO) have been deposited in the PDB (http://wwpdb.org/). The amino acid sequence of this protein can be accessed through NCBI Protein Database under NCBI Accession QDD67358 (http://www.ncbi.nlm.nih.gov/sites/gquery).

## Supporting information

This article contains supporting information (Table S1 and Figs. S1-S5) (24, 25, 26, 27).

## Conflict of interest

The authors declare that they have no conflicts of interest with the contents of this article.

## References

[1] Fenoradosoa T.A., Ali G., Delattre C., Laroche C., Petit E., Wadouachi A. (2010). Extraction and characterization of an alginate from the brown seaweed *Sargassum turbinarioides* Grunow. J. Appl. Phycol..

[2] Gacesa P. (1988). Alginates. Carbohyd Polym..

[3] Lee K.Y., Mooney D.J. (2012). Alginate: properties and biomedical applications. Prog. Polym. Sci..

[4] Zhang L., Li X., Zhang X., Li Y., Wang L. (2021). Bacterial alginate metabolism: an important pathway for bioconversion of brown algae. Biotechnol. Biofuels.

[5] Vasudevan U.M., Lee O.K., Lee E.Y. (2021). Alginate derived functional oligosaccharides: recent developments, barriers, and future outlooks. Carbohyd Polym..

[6] Xing M., Cao Q., Wang Y., Xiao H., Zhao J., Zhang Q. (2020). Advances in research on the bioactivity of alginate oligosaccharides. Mar. Drugs.

[7] Zhang C.H., Li M.X., Rauf A., Khalil A.A., Shan Z.G., Chen C.Y. (2021). Process and applications of alginate oligosaccharides with emphasis on health beneficial perspectives. Crit. Rev. Food Sci..

[8] Xiong B.H., Liu M., Zhang C., Hao Y.N., Zhang P.F., Chen L. (2020). Alginate oligosaccharides enhance small intestine cell integrity and migration ability. Life Sci..

[9] Drula E., Garron M.L., Dogan S., Lombard V., Henrissat B., Terrapon N. (2021). The carbohydrate-active enzyme database: functions and literature. Nucl. Acids Res..

[10] Dong S., Wei T.D., Chen X.L., Li C.Y., Wang P., Xie B.B. (2014). Molecular insight into the role of the N-terminal extension in the maturation, substrate recognition, and catalysis of a bacterial alginate lyase from polysaccharide lyase family 18. J. Biol. Chem..

[11] Ji S., Dix S.R., Aziz A.A., Sedelnikova S.E., Baker P.J., Rafferty J.B. (2019). The molecular basis of endolytic activity of a multidomain alginate lyase from *Defluviitalea phaphyphila*, a representative of a new lyase family, PL39. J. Biol. Chem..

[12] Li S., Yang X., Bao M., Wu Y., Yu W., Han F. (2015). Family 13 carbohydrate-binding module of alginate lyase from *Agarivorans sp.* L11 enhances its catalytic efficiency and thermostability, and alters its substrate preference and product distribution. FEMS Microbiol. Lett..

[13] Boraston A.B., Bolam D.N., Gilbert H.J., Davies G.J. (2004). Carbohydrate-binding modules: fine-tuning polysaccharide recognition. Biochem. J..

[14] Gilbert H.J., Knox J.P., Boraston A.B. (2013). Advances in understanding the molecular basis of plant cell wall polysaccharide recognition by carbohydrate-binding modules. Curr. Opin. Struct. Biol..

[15] Montanier C., van Bueren A.L., Dumon C., Flint J.E., Correia M.A., Prates J.A. (2009). Evidence that family 35 carbohydrate binding modules display conserved specificity but divergent function. Proc. Natl. Acad. Sci. U. S. A..

[16] Armenta S., Moreno-Mendieta S., Sanchez-Cuapio Z., Sanchez S., Rodriguez-Sanoja R. (2017). Advances in molecular engineering of carbohydrate-binding modules. Proteins.

[17] Shoseyov O., Shani Z., Levy I. (2006). Carbohydrate binding modules: biochemical properties and novel applications. Microbiol. Mol. Biol. Rev..

[18] Lyu Q., Zhang K., Zhu Q., Li Z., Liu Y., Fitzek E. (2018). Structural and biochemical characterization of a multidomain alginate lyase reveals a novel role of CBM32 in CAZymes. Biochim. Biophys. Acta Gen. Subj..

[19] Sim P.F., Furusawa G., Teh A.H. (2017). Functional and structural studies of a multidomain alginate lyase from *Persicobacter sp.* CCB-QB2. Sci. Rep..

[20] Teh A.H., Sim P.F., Hisano T. (2020). Structural basis for binding uronic acids by family 32 carbohydrate-binding modules. Biochem. Biophys. Res. Commun..

[21] Jamal-Talabani S., Boraston A.B., Turkenburg J.P., Tarbouriech N., Ducros V.M., Davies G.J. (2004). *Ab initio* structure determination and functional characterization of CBM36; a new family of calcium-dependent carbohydrate binding modules. Structure.

[22] Holm L. (2020). Using dali for protein structure comparison. Met. Mol. Biol..

[23] Adindla S., Inampudi K.K., Guruprasad K., Guruprasad L. (2004). Identification and analysis of novel tandem repeats in the cell surface proteins of archaeal and bacterial genomes using computational tools. Comp. Funct. Genom..

[24] Madeira F., Park Y.M., Lee J., Buso N., Gur T., Madhusoodanan N. (2019). The EMBL-EBI search and sequence analysis tools APIs in 2019. Nucl. Acids Res..

[25] Ashkenazy H., Abadi S., Martz E., Chay O., Mayrose I., Pupko T. (2016). ConSurf 2016: an improved methodology to estimate and visualize evolutionary conservation in macromolecules. Nucl. Acids Res..

[26] Celniker G., Nimrod G., Ashkenazy H., Glaser F., Martz E., Mayrose I. (2013). ConSurf: using evolutionary data to raise testable hypotheses about protein function. Isr. J. Chem..

[27] Ashkenazy H., Erez E., Martz E., Pupko T., Ben-Tal N. (2010). ConSurf 2010: calculating evolutionary conservation in sequence and structure of proteins and nucleic acids. Nucl. Acids Res..

[28] Jurrus E., Engel D., Star K., Monson K., Brandi J., Felberg L.E. (2018). Improvements to the APBS biomolecular solvation software suite. Protein Sci..

[29] Doxey A.C., Cheng Z., Moffatt B.A., McConkey B.J. (2010). Structural motif screening reveals a novel, conserved carbohydrate-binding surface in the pathogenesis-related protein PR-5d. BMC Struct. Biol..

[30] Baek M., DiMaio F., Anishchenko I., Dauparas J., Ovchinnikov S., Lee G.R. (2021). Accurate prediction of protein structures and interactions using a three-track neural network. Science.

[31] Ficko-Blean E., Gregg K.J., Adams J.J., Hehemann J.H., Czjzek M., Smith S.P. (2009). Portrait of an enzyme, a complete structural analysis of a multimodular *beta*-N-acetylglucosaminidase from *Clostridium perfringens*. J. Biol. Chem..

[32] Herve C., Rogowski A., Blake A.W., Marcus S.E., Gilbert H.J., Knox J.P. (2010). Carbohydrate-binding modules promote the enzymatic deconstruction of intact plant cell walls by targeting and proximity effects. Proc. Natl. Acad. Sci. U. S. A..

[33] Ji S.Q., Wang B., Lu M., Li F.L. (2016). *Defluviitalea phaphyphila sp. nov*., a novel thermophilic bacterium that degrades brown algae. Appl. Environ. Microbiol..

[34] McCartney L., Blake A.W., Flint J., Bolam D.N., Boraston A.B., Gilbert H.J. (2006). Differential recognition of plant cell walls by microbial xylan-specific carbohydrate-binding modules. Proc. Natl. Acad. Sci. U. S. A..

[35] Franklin M.J., Nivens D.E., Weadge J.T., Howell P.L. (2011). Biosynthesis of the *Pseudomonas aeruginosa* extracellular polysaccharides, alginate, Pel, and Psl. Front. Microbiol..

[36] Wilkins M.R., Gasteiger E., Bairoch A., Sanchez J.C., Williams K.L., Appel R.D. (1999). Protein identification and analysis tools in the ExPASy server. Met. Mol. Biol..

[37] Hellman L.M., Fried M.G. (2007). Electrophoretic mobility shift assay (EMSA) for detecting protein-nucleic acid interactions. Nat. Protoc..

[38] Altschul S.F., Gish W., Miller W., Myers E.W., Lipman D.J. (1990). Basic local alignment search tool. J. Mol. Biol..

[39] Kumar S., Stecher G., Tamura K. (2016). MEGA7: molecular evolutionary genetics analysis version 7.0 for bigger datasets. Mol. Biol. Evol..

[40] Evans P. (2006). Scaling and assessment of data quality. Acta Crystallogr. D Biol. Crystallogr..

[41] Evans P.R., Murshudov G.N. (2013). How good are my data and what is the resolution?. Acta Crystallogr. D.

[42] Kabsch W. (2010). Xds. Acta Crystallogr. D Biol. Crystallogr..

[43] Tickle I.J., Flensburg C., Keller P., Paciorek W., Sharff A., Vonrhein C. (2020).

[44] Vonrhein C., Flensburg C., Keller P., Sharff A., Smart O., Paciorek W. (2011). Data processing and analysis with the autoPROC toolbox. Acta Crystallogr. D Biol. Crystallogr..

[45] Winn M.D., Ballard C.C., Cowtan K.D., Dodson E.J., Emsley P., Evans P.R. (2011). Overview of the CCP4 suite and current developments. Acta Crystallogr. Sect. D-Struct. Biol..

[46] Sheldrick G.M. (2008). A short history of SHELX. Acta Crystallogr. A..

[47] Cowtan K. (2008). Fitting molecular fragments into electron density. Acta Crystallogr. D Biol. Crystallogr..

[48] Emsley P., Lohkamp B., Scott W.G., Cowtan K. (2010). Features and development of Coot. Acta Crystallogr. D Biol. Crystallogr..

[49] Murshudov G.N., Skubak P., Lebedev A.A., Pannu N.S., Steiner R.A., Nicholls R.A. (2011). REFMAC5 for the refinement of macromolecular crystal structures. Acta Crystallogr. D Biol. Crystallogr..

[50] Williams C.J., Headd J.J., Moriarty N.W., Prisant M.G., Videau L.L., Deis L.N. (2018). MolProbity: more and better reference data for improved all-atom structure validation. Protein Sci..

[51] The PyMOL Molecular Graphics System, version 2.4.0a0 open-source. Schrödinger, LLC.

[52] Morris G.M., Huey R., Lindstrom W., Sanner M.F., Belew R.K., Goodsell D.S. (2009). AutoDock4 and AutoDockTools4: automated docking with selective receptor flexibility. J. Comput. Chem..

[53] Trott O., Olson A.J. (2010). AutoDock Vina: improving the speed and accuracy of docking with a new scoring function, efficient optimization, and multithreading. J. Comput. Chem..

[54] Mi L.Z., Brown C.T., Gao Y., Tian Y., Le V.Q., Walz T. (2015). Structure of bone morphogenetic protein 9 procomplex. Proc. Natl. Acad. Sci. U. S. A..

[55] Wang X., Fischer G., Hyvonen M. (2016). Structure and activation of pro-activin A. Nat. Commun..

[56] Morgan J.L., McNamara J.T., Fischer M., Rich J., Chen H.M., Withers S.G. (2016). Observing cellulose biosynthesis and membrane translocation in crystallo. Nature.

[57] Kuhn P., Guan C., Cui T., Tarentino A.L., Plummer T.H., Van Roey P. (1995). Active site and oligosaccharide recognition residues of peptide-N4-(N-acetyl-*beta*-D-glucosaminyl)asparagine amidase F. J. Biol. Chem..

[58] Suzuki N., Fujimoto Z., Kim Y.M., Momma M., Kishine N., Suzuki R. (2014). Structural elucidation of the cyclization mechanism of *alpha*-1,6-glucan by *Bacillus circulans* T-3040 cycloisomaltooligosaccharide glucanotransferase. J. Biol. Chem..

[59] Fujimoto Z., Suzuki N., Kishine N., Ichinose H., Momma M., Kimura A. (2017). Carbohydrate-binding architecture of the multi-modular *alpha*-1,6-glucosyltransferase from *Paenibacillus sp.* 598K, which produces *alpha*-1,6-glucosyl-*alpha*-glucosaccharides from starch. Biochem. J..

[60] Henshaw J., Horne-Bitschy A., van Bueren A.L., Money V.A., Bolam D.N., Czjzek M. (2006). Family 6 carbohydrate binding modules in *beta*-agarases display exquisite selectivity for the non-reducing termini of agarose chains. J. Biol. Chem..

