# Supplementary material for: Identification and structural analysis of a carbohydrate-binding module specific to alginate, a representative of a new family, CBM96
Source: J Biol Chem. 2022 Dec 31;299(2):102854. doi: 10.1016/j.jbc.2022.102854 (PMC9971899; doi:10.1016/j.jbc.2022.102854)
Supplement: Supporting information [file mmc1.docx]

**Supporting Information**

**Table S1. Primers for recombinant enzymes in this work.**

|  | **Primers name** | **Sequences (5＇to 3＇)** |
| --- | --- | --- |
| TM6 | TM6-fwd | gtatggctagcgaattggccaaagaatttgcccaaggagaag |
|  | TM6-rev | agcagccggatcgttgagctctataatacaatttcttcagttttcc |
| TM6-N1 | N1-fwd | gtatggctagcgaattggccaaagaatttgcccaaggagaag |
|  | N1-rev | agcagccggatcgttgagctaactactgcttctaaggaa |
| TM6-N2 | N2-fwd | gtatggctagcgaattggccataccaactggagaaggtag |
|  | N2-rev | agcagccggatcgttgagctctataatacaatttcttcagttttcc |
| TM6-N3 | N3-fwd | gtatggctagcgaattggccgagcctatgactaatgata |
|  | N3-rev | agcagccggatcgttgagctctataatacaatttcttcagttttcc |
| TM6-N4 | N4-fwd | gtatggctagcgaattggccccaacagatgatactta |
|  | N4-rev | agcagccggatcgttgagctctataatacaatttcttcagttttcc |
| TM6-N5 | N5-fwd | gtatggctagcgaattggcctcttcaactgtagatagt |
|  | N5-rev | agcagccggatcgttgagctctataatacaatttcttcagttttcc |
| TM6-N6 | N6-fwd | gtatggctagcgaattggccccaacagatgatactta |
|  | N6-rev | agcagccggatcgttgagctttcttgagttgaacgg |
| N4-K10A | K10A-fwd  K10A-rev | ctatctacagttgaagatgcattttcaacataagtatcatc  gatgatacttatgttgaaaatgcatcttcaactgtagatag |
| N4-K22A | K22A-fwd  K22A-rev | agtaattttgcaacatcagcacaattgaagtttaaagga  tcctttaaacttcaattgtgctgatgttgcaaaattact |
| N4-Q23A | Q23A-fwd  Q23A-rev | taattttgcaacatcaaaagcattgaagtttaaaggaact  agttcctttaaacttcaatgcttttgatgttgcaaaatta |
| N4-K25A | K25A-fwd  K25A-rev | cctttagaagttcctttaaatgccaattgttttgatgttgc  gcaacatcaaaacaattggcatttaaaggaacttctaaagg |
| N4-K27A | K27A-fwd  K27A-rev | ctacctttagaagttcctgcaaacttcaattgttttgat  atcaaaacaattgaagtttgcaggaacttctaaaggtag |
| N4-K31A | K31A-fwd  K31A-rev | aagtttaaaggaacttctgcaggtagtgacgatagaa  ttctatcgtcactacctgcagaagttcctttaaactt |
| N4-D34A | D34A-fwd  D34A-rev | gaacttctaaaggtagtgcagatagaataggatatttaa  ttaaatatcctattctatctgcactacctttagaagttc |
| N4-R36E | R36E-fwd  R36E-rev | gtcaaattttaaatatcctatctcatcgtcactacctttag  ctaaaggtagtgacgatgagataggatatttaaaatttgac |
| N4-N157A | N157A-fwd  N157A-rev | ctgaccaagatcaaaatgcagcttatggttggttcc  ggaaccaaccataagctgcattttgatcttggtcag |
| N4-Y159A | Y159A-fwd | gaccaagatcaaaataatgctgctggttggttccgttcaac |
|  | Y159A-rev | gttgaacggaaccaaccagcagcattattttgatcttggtc |


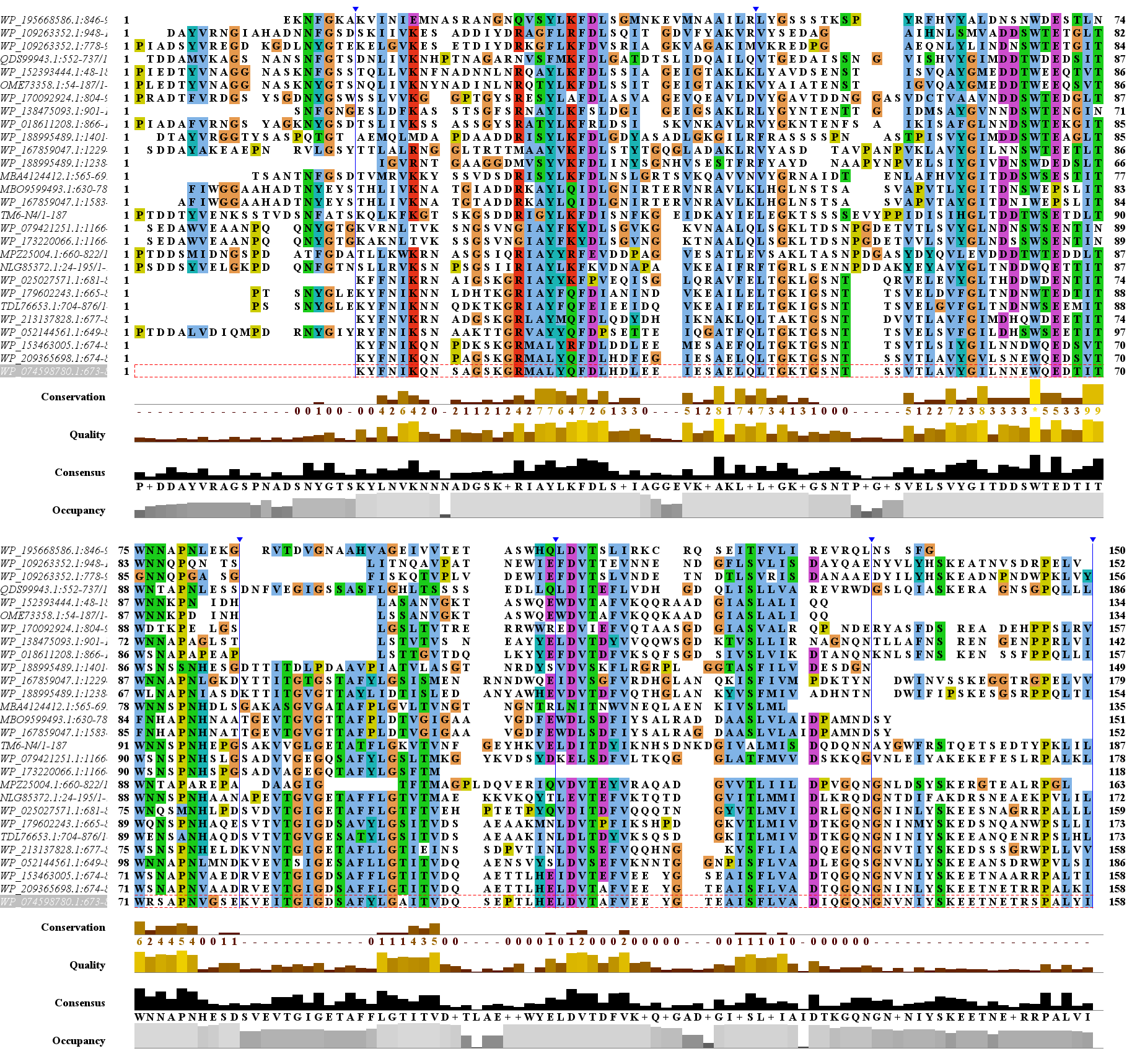


**Figure S1.** Amino acid sequence alignment of TM6-N4 and its closest relatives by Clustal Omega (EMBL-EBI) (1). The depth of blue was used to show the percentage identity. Conservation, consensus, and occupancy were shown as histograms below the sequences.

**
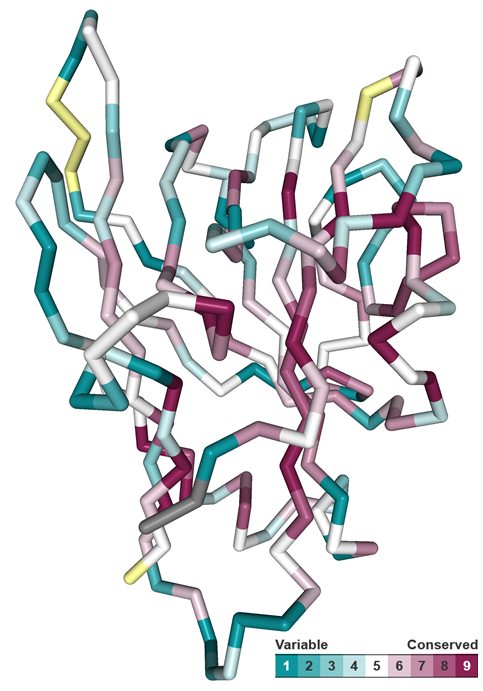
**

**Figure S2.** ConSurf (<https://consurf.tau.ac.il/consurf_index.php>) (2-4) identifying evolutionary conservation profiles for TM6-N4 from a sample of 150 sequences that represent the list of homologues to the query. High conservation grades colored in magenta.

**
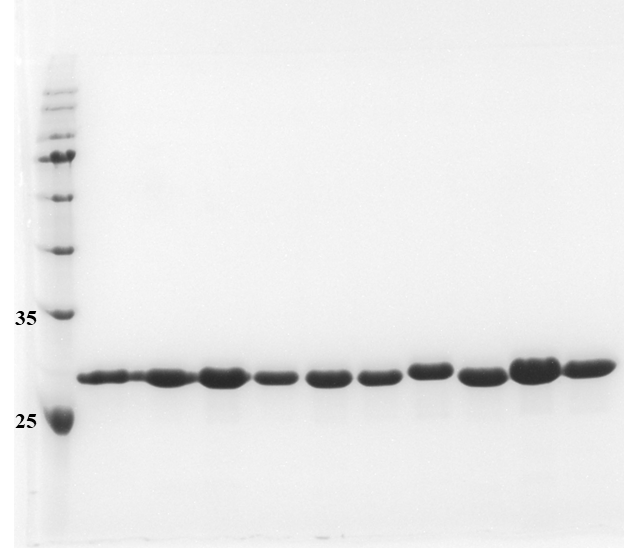
**

**Figure S3.** SDS-PAGE of site-directed mutants of TM6-N4. Lane 1 to lane 11 represent marker, K10A, K22A, Q23A, K25A, K27A, K31A, D34A, R36E, N157A and Y159A respectively.





**Figure S4.** Circular dichroism (CD) spectra of wild-type and the site-directed mutants of TM6-N4 used in the manuscript.


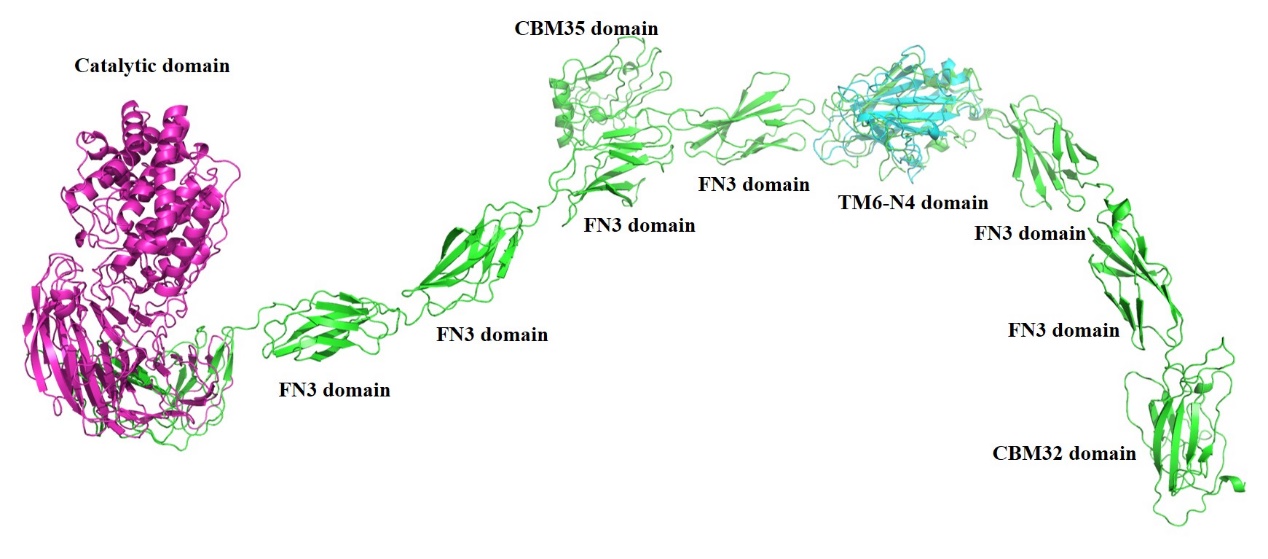


**Figure S5.** Portrait of multidomain alginate lyase Dp0100. The green cartoon showed the structure of Dp0100-1183 (Ala617-Gln1799) predicated by RoseTTAFold. The magenta cartoon showed the structure of the catalytic domain of Dp0100 (PDB code 6JP4), while the cyan cartoon showed the structure of TM6-N4 (PDB code 7VBO) in this study. The structures are aligned by using PyMOL.

**References**

1. Madeira F., Park Y.M., Lee J., Buso N., Gur T., Madhusoodanan N., The EMBL-EBI search and sequence analysis tools APIs in 2019 Nucl. Acids Res. 2019 W636-W641
2. Ashkenazy H., Abadi S., Martz E., Chay O., Mayrose I., Pupko T., ConSurf 2016: an improved methodology to estimate and visualize evolutionary conservation in macromolecules Nucl. Acids Res. 2016 W344-W350
3. Celniker G., Nimrod G., Ashkenazy H., Glaser F., Martz E., Mayrose I., ConSurf: using evolutionary data to raise testable hypotheses about protein function Isr. J. Chem. 2013 199-206
4. Ashkenazy H., Erez E., Martz E., Pupko T., Ben-Tal N., ConSurf 2010: calculating evolutionary conservation in sequence and structure of proteins and nucleic acids Nucl. Acids Res. 2010 W529-W533
